# Is cytoreductive surgery and hyperthermic intraperitoneal chemotherapy indicated in hepatobiliary malignancies?

**DOI:** 10.1186/s12957-020-01898-5

**Published:** 2020-06-11

**Authors:** Natasha Leigh, Daniel Solomon, Eric Pletcher, Daniel M. Labow, Deepa R. Magge, Umut Sarpel, Benjamin J. Golas

**Affiliations:** Division of Surgical Oncology, Icahn School of Medicine at Mount Sinai St. Luke’s West Hospital, 425 West 59th Street, Suite 7B, New York, NY 10019 USA

**Keywords:** Peritoneal carcinomatosis, Hyperthermic intraperitoneal chemotherapy, Cytoreductive surgery, Hepatocellular carcinoma, Hepatobiliary malignancy

## Abstract

**Background:**

Hepatopancreaticobiliary malignancies with peritoneal carcinomatosis exhibit poor survival with current therapies: hepatocellular carcinoma 11 months with sorafenib, and pancreaticobiliary 9–14 months with systemic chemotherapy. However, limited data exist on the utility of cytoreductive surgery and hyperthermic intraperitoneal chemotherapy in these patients.

**Methods:**

We retrospectively reviewed our institutional hepatopancreaticobiliary malignancies with peritoneal carcinomatosis which underwent cytoreductive surgery and hyperthermic intraperitoneal chemotherapy from 2007 to 2017 and analyzed perioperative and oncologic outcomes.

**Results:**

Seventeen patients were included: 9 hepatocellular carcinoma, 8 pancreaticobiliary (4 cholangiocarcinoma, 3 gallbladder, 1 pancreatic). Peritoneal cancer index, number of organs resected, completeness of cytoreduction, and 30-day morbidity were equivalent. Hepatocellular carcinoma received significantly less neoadjuvant therapy (11%, *p* = 0.008), though adjuvant therapy rates were similar. At a median follow-up of 15 months, progression-free survival was similar amongst all cohorts. However, overall survival was longer in hepatocellular carcinoma (42 months vs. cholangiocarcinoma 19 months, gallbladder 8 months, pancreatic 15 months, *p* = 0.206) with 59% 3-year overall survival (vs. 0% cholangiocarcinoma, 0% gallbladder, 0% pancreatic).

**Conclusions:**

Cytoreductive surgery and hyperthermic intraperitoneal chemotherapy may offer a survival benefit in select hepatocellular carcinoma patients with peritoneal carcinomatosis, though has dubious utility in pancreaticobiliary malignancies.

## Background

Hepatopancreaticobiliary (HPB) malignancies are the twelfth most common cancers in the USA, but the third most common cause of cancer-related mortality [1]. Their aggressive nature and vague symptomatology frequently lead to delayed diagnosis, and the majority are locally advanced or metastatic at presentation rendering them inoperable.

In the setting of peritoneal carcinomatosis (PC), palliative chemotherapy remains the mainstay of treatment [2], though it offers limited long-term survival benefits. Hyperthermic intraperitoneal chemotherapy (HIPEC) and cytoreductive surgery (CRS) have been shown to improve prognosis in patients with PC from colorectal malignancies [3, 4], appendiceal pseudomyxoma peritonei [5], and primary peritoneal mesothelioma [6]. A few small studies have investigated the role of CRS/HIPEC in hepatocellular carcinoma (HCC) [7–11] with some suggestion of prognostic benefit; however, data is even more limited for pancreaticobiliary malignancies (cholangiocarcinoma (CCa), gallbladder cancer (GBC), and pancreatic cancer (PCa)) [12–14].

The purpose of this study was to analyze short-term perioperative outcomes and long-term survival data in order to determine the utility of CRS and HIPEC in HPB malignancies with PC. We hypothesized that HCC with PC would exhibit at least equivalent if not longer survival compared with sorafenib, and pancreaticobiliary malignancies with PC would have no survival benefit after CRS and HIPEC compared with systemic chemotherapy.

## Methods

This is a retrospective study of 17 consecutive patients who underwent attempted CRS/HIPEC for PC secondary to HPB primaries at a single institution between August 2007 and April 2017. Preoperative evaluation included routine laboratory work, radiographic staging, and assessment of physiologic fitness for CRS/HIPEC. Patients with any radiographic evidence of metastatic disease outside of the peritoneal cavity or those whose procedures were aborted were excluded. All patients were discussed in a multidisciplinary tumor board prior to surgery. For HCC, resection was only considered in Child’s A disease without clinical evidence of portal hypertension (platelet count < 100,000/μl, imaging findings of splenomegaly, varices, or ascites) provided patients had adequate future liver remnant (FLR). All procedures were performed by surgical oncologists familiar with CRS/HIPEC. This study was approved by the Mt. Sinai School of Medicine institutional review board.

### Surgical procedure

CRS/HIPEC was performed in a standard fashion as previously described [15]. Diagnostic laparoscopy was performed in all cases to assess the feasibility of cytoreduction prior to HIPEC. The procedure was aborted at the discretion of the operating surgeon if the tumor burden was deemed too bulky to attempt cytoreduction. The peritoneal cancer index (PCI) was calculated prior to operative debulking [16], and the completeness of cytoreduction (CC) score recorded at the conclusion of the procedure. Complete cytoreduction was defined as CC-0 (no macroscopic disease) or CC-1 (residual tumor implants < 2.5 mm). Incomplete cytoreduction was defined as CC-2 (residual tumor implants between 2.5 and 2.5 cm in diameter) or CC-3 (residual tumor implants greater than 2.5 cm in diameter). All patients who underwent HIPEC received 40 mg of mitomycin C at 42 °C for 90 min (30 mg for 60 min followed by an additional 10 mg for the final 30 min). Creation of anastomoses was performed after the completion of HIPEC. Major perioperative complications were graded according to the Clavien-Dindo classification system (III–V) [17] and defined as occurring within 30 days of CRS/HIPEC.

### Data collection

Detailed clinicopathologic data encompassing the preoperative, intraoperative, and postoperative course was collected retrospectively and maintained within a prospectively managed database.

### Outcomes

Primary endpoints were progression-free survival (PFS) and overall survival (OS). OS was defined as time from surgery to death or last follow-up. PFS was defined as time from surgery to disease progression or relapse (diagnosed on imaging or re-operation). Postoperatively, patients were followed with serial contrast-enhanced cross-sectional imaging, although there were no strict institutional protocols in place. Secondary endpoints were rate of complete cytoreduction, estimated blood loss (EBL), operative time, length of stay (LOS), and major perioperative morbidity.

### Statistical analysis

All statistical analyses were performed using the SAS® software, version 9.4. Categorical variables were compared using Fisher’s exact tests and are reported as totals with percentages. Continuous variables were compared using ANOVA tests and are reported as median values with interquartile ranges (IQR). Normality of distribution was assessed using Shapiro-Wilk tests. Kaplan-Meier estimates were used to analyze PFS and OS and survival curves calculated using the log-rank test. Cox-proportional hazards models were used to create multivariate models for factors predictive of PFS and OS and are reported as hazard ratios (HR) with 95% confidence intervals (CI). A *p* value of < 0.05 was considered to be statistically significant.

## Results

### Patient characteristics

A total of 17 patients with PC secondary to primary HPB malignancies underwent attempted CRS/HIPEC. There were 9 HCC primaries and 8 pancreaticobiliary primaries (4 CCa (3 intrahepatic, 1 hilar), 3 GBC (adenocarcinoma), and 1 PCa (mucin-producing ductal adenocarcinoma)). Clinicopathologic characteristics are reported in Table 1. The cohorts were similar in all preoperative factors, except that significantly more pancreaticobiliary patients underwent neoadjuvant chemotherapy (CCa *n* = 2, 50%; GBC *n* = 3, 100%; PCa *n* = 1, 100% versus HCC *n* = 1, 11%, *p* = 0.008). The most frequent regimens used were gemcitabine for CCa and GBC. The PCa patient received gemcitabine. The regimen was unknown for the one HCC patient who received neoadjuvant chemotherapy. Overall, the majority of patients underwent CRS/HIPEC for metachronous lesions (HCC *n* = 8, 89%; CCa *n* = 2, 50%; GBC *n* = 3, 100%), although the PCa patient had synchronous PC.

**Table 1:** Clinicopathologic characteristics of the four cohorts

Characteristic	HCC (*n* = 9)	CCa (*n* = 4)	GBC (*n* = 3)	PCa (*n* = 1)	*p* value
Age at surgery, years	58 [49–62]	67 [60–72]	49 [38–66]	64 [64]	0.438
Gender (male/female)	8 (89)/1 (11)	1 (25)/3 (75)	2 (67)/1 (33)	1 (100)/0 (0)	0.082
ASA score III/IV	9 (100)	3 (75)	3 (100)	1 (100)	0.559
Prior abdominal surgery	6 (67)	3 (75)	3 (100)	0 (0)	0.626
Synchronous/metachronous PC	1 (11)/8 (89)	2 (50)/2 (50)	0 (0)/3 (100)	1 (100)/0 (0)	0.082
Neoadjuvant chemotherapy	1 (11)	2 (50)	3 (100)	1 (100)	0.012*

*HCC* hepatocellular carcinoma, *CCa* cholangiocarcinoma, *GBC* gallbladder cancer, *PCa* pancreatic cancer, *PC* peritoneal carcinomatosis

* *p*<0.05

### Perioperative outcomes

All 17 patients underwent both CRS and HIPEC (Table 2). For the entire cohort, the median PCI was 10 (IQR 6–18). Complete cytoreduction was achieved in the majority of patients in the HCC (*n* = 7, 78%), CCa (*n* = 4, 100%), and PCa (*n* = 1, 100%) cohorts. The GBC cohort only achieved complete cytoreduction in one third of patients (*n* = 1). There was no significant difference between the cohorts in terms of PCI, number of organs resected, CC score, EBL, or OR time. Perioperative outcomes were also similar (LOS, ICU stay, perioperative major morbidity, perioperative mortality). The use of adjuvant chemotherapy was similar in both cohorts. The most frequent regimens used were gemcitabine and FOLFIRI for CCa, and gemcitabine-oxaliplatin for GBC. Sorafenib was the agent in all 7 of the HCC patients who received adjuvant therapy. The regimen was unknown for the one PCa patient who received adjuvant chemotherapy. Postoperative serial imaging for HCC patients included a CT with or without MRI every 3–4 months for up to 5 years. The pancreatic patient had no follow-up imaging recorded. Two of the patients with gallbladder cancer underwent serial CT scans every 2–3 months; one patient had no postoperative imaging. All 4 of the CCa patients had serial CT scans every 3–4 months; 2 of those patients had one or more PET/CT scans.

**Table 2:** Perioperative outcomes of the four cohorts

Value	HCC (*n* = 9)	CCa (*n* = 4)	GBC (*n* = 3)	PCa (*n* = 1)	*p* value
PCI	12 [6–16]	7 [4–13]	21 [7–27]	10 [10]	0.538
Number of organs resected	2 [1–5]	2 [1–5]	4 [4–9]	6 [6]	0.626
CC score					0.668
CC-0/1	7 (78)	4 (100)	1 (33)	1 (100)	
CC-2/3	2 (22)	0 (0)	2 (67)	0 (0)	
EBL, cc	500 [100–700]	450 [225–750]	600 [200–2000]	150 [150]	0.780
OR time, min	305 [250–388]	304 [231–327]	345 [302–385]	358 [358]	0.795
LOS, days	5 [5–9]	6 [6–8]	7 [5–8]	3 [3]	0.833
ICU stay	4 (44)	0 (0)	1 (33)	0 (0)	0.430
Perioperative major morbidity	1 (11)	0 (0)	1 (33)	0 (0)	0.471
Perioperative mortality	1 (11)	0 (0)	0 (0)	0 (0)	0.815
Adjuvant chemotherapy	7 (78)	4 (100)	3 (100)	1 (100)	0.569
Follow-up time, months	23 [3–42]	19 [15–21]	2 [0–13]	15 [15]	0.493

*HCC* hepatocellular carcinoma, *CCa* cholangiocarcinoma, *GBC* gallbladder cancer, *PCa* pancreatic cancer, *CRS* cytoreductive surgery, *HIPEC* hyperthermic intraperitoneal chemotherapy, *PCI* peritoneal cancer index, *CC* completeness of cytoreduction, *EBL* estimated blood loss, *OR* operating room, *LOS* length of stay, *ICU* intensive care unit

### Survival outcomes

**Fig. 1:**
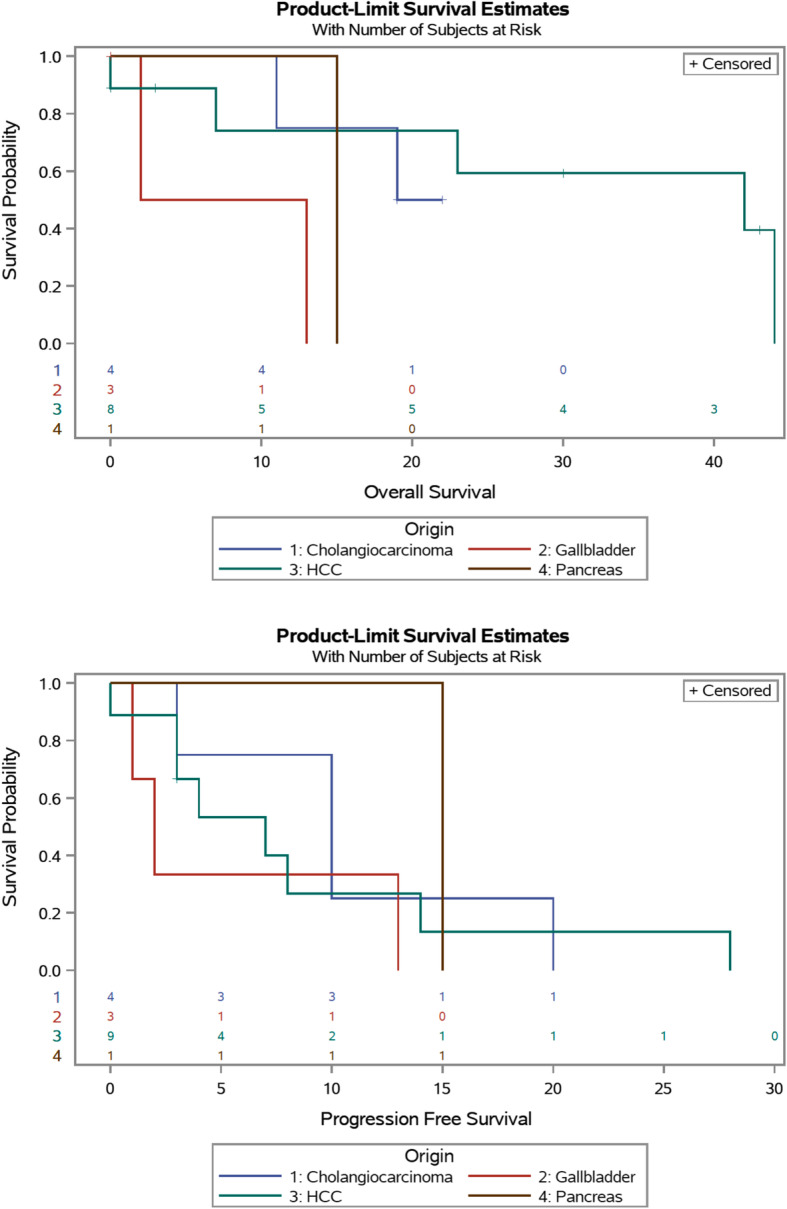
Kaplan-Meier curves for survival. **a** Overall survival for the four cohorts. **b** Progression-free survival for the four cohorts

Table 3 displays survival outcomes. Median follow-up time was 15 months. Median OS for the entire cohort was 23 months with 1-year and 3-year OS rates of 73% and 41%, respectively. There was a notable trend towards longer OS in the HCC cohort (median 42 months) compared to the other cohorts, though statistical significance was not reached, *p* = 0.188 (Fig. 1a). Of the pancreaticobiliary malignancies, the longest median OS was seen in CCa (19 months), though this was still considerably shorter than in HCC. Of note, the 3 patients with intrahepatic CCa had a much longer median OS (19 months) than the 1 patient with hilar CCa (11 months). Median PFS for the entire cohort was 8 months, and there was no statistically significant difference between the cohorts, *p* = 0.550 (Fig. 1b). All patients experienced tumor recurrence by 3 years postoperatively. The shortest median PFS was in GBC (2 months), and the longest was in PCa (15 months). On multivariate analysis, age at surgery (HR 1.13, CI 1.01–1.26, *p* = 0.027) and PCI (HR 1.24, CI 1.05–1.47, *p* = 0.011) were independent predictors of OS. There were no independent predictors of PFS.

**Table 3:** Survival data for the four cohorts

	Overall survival			*p* value	Progression-free survival			*p* value
	Median, months	1-year (%)	3-year (%)		Median, months	1-year (%)	3-year (%)	
HCC (*n* = 9)	42 [7–44]	5 (74)	3 (59)	0.188	7 [3–14]	2 (27)	0 (0)	0.550
CCa (*n* = 4)	19 [15–NR]	3 (75)	0 (0)		10 [7–15]	1 (25)	0 (0)	
GBC (*n* = 3)	8 [2–13]	1 (50)	0 (0)		2 [1–13]	1 (33)	0 (0)	
PCa (*n* = 1)	15 [N/A]	1 (100)	0 (0)		15 [N/A]	1 (100)	0 (0)	

*HCC* hepatocellular carcinoma, *CCa* cholangiocarcinoma, *GBC* gallbladder cancer, *PCa* pancreatic cancer, *NR* not reached

## Discussion

### Pancreaticobiliary malignancies

#### Cholangiocarcinoma

In the setting of CCa with metastatic disease, the best supportive care offers a median survival of 2.5 months [18]. Though the addition of gemcitabine/capecitabine-based palliative chemotherapeutic regimens has improved survival to 9.3–14 months [19, 20], it still remains poor. To the best of our knowledge, to date, there are only two case reports in the literature of metastatic CCa managed with CRS/HIPEC. Golse et al. reported on a retrospective series of iterative CRS/HIPEC procedures [13]. One patient with CCa was treated twice with CRS/HIPEC for tumor recurrence limited to the peritoneal cavity. Though the specific PCI for this patient was not reported, they required limited debulking (omentectomy and peritonectomy) and complete cytoreduction was achieved. The HIPEC agents used were mitomycin C and cisplatin. Neither the specific morbidity nor the survival data was available. The second case, reported by Brandl et al. [14], underwent a single CRS/HIPEC procedure with a PCI of 16 and achieved CC-0. The HIPEC agent was not stated. PFS was 12.1 months, and OS was 12.7 months. Neither study reported if the malignancies originated from the intra- or extrahepatic biliary tree. In our study, all four patients with CCa (three intrahepatic, one hilar) underwent a single CRS/HIPEC procedure; 50% were for metachronous PC. The median PCI was 7, all patients achieved complete cytoreduction (requiring 1–6 organ resections), and mitomycin C was used as the HIPEC agent in all procedures. Two patients underwent neoadjuvant chemotherapy, and all underwent adjuvant chemotherapy with gemcitabine or FOLFIRINOX. No patients experienced major perioperative morbidity. Median PFS was 10 months, and median OS was 19 months. Hilar CCa had a shorter OS (11 months) and PFS (3 months) compared to intrahepatic origin.

#### Gallbladder adenocarcinoma

Advanced GBC has a 5-year survival rate of less than 5% [21]. Phase II trials with gemcitabine-based chemotherapeutic regimens have demonstrated survival benefits: PFS 6–9 months and OS 9.8–14 months [22]. The ABC-02 phase III trial by Valle et al. reported significant superiority of gemcitabine/cisplatin over gemcitabine alone with improved median PFS (8 versus 5 months) and median OS (11.7 versus 8.1 months); thus, gemcitabine-cisplatin is now the standard of care for advanced biliary tract cancers [23]. However, there is limited data regarding the role of CRS/HIPEC in GBC. Most reports of debulking only include patients with locally advanced, non-metastatic GBC [24, 25], and there are only very few discussing outcomes of metastasectomy [26, 27]. A 2017 study by Park et al. reviewed 19 patients with metastatic biliary tract cancers; 7 had GBC with PC (3 were metachronous disease) [28]. After metastasectomy, only two achieved R0 and three achieved R1. Median PFS and OS were 8.5 and 16.6 months, respectively, longer in patients with metachronous metastases and fewer organs resected. There is only one study in the literature examining CRS/HIPEC for GBC. Randle et al. reviewed 5 patients undergoing 6 procedures for GBC with PC [12]. All patients had low PCI scores and achieved complete cytoreduction. Major perioperative morbidity was reported in 17% of patients. Median OS was 22.4 months, and 3-year OS was 30%. In our study, there were three patients with GBC who underwent CRS/HIPEC for metachronous metastases. The median PCI was 21, and only 1 patient achieved complete cytoreduction; the remaining two had a CC-2 score. All patients underwent neoadjuvant and adjuvant chemotherapy with gemcitabine-based regimens. One patient experienced major morbidity with wound complications requiring reoperation. PFS was 13 months for the patient who achieved CC-0, and median OS was 7.5 months (13 months for the CC-0 patient).

#### Pancreatic adenocarcinoma

Over the last few decades, the approach to managing PCa has undergone a paradigm shift. It has largely become a non-operative disease treated with chemotherapy owing to the morbidity of the procedures as well as the frequent occurrence of occult metastases at diagnosis. Postoperative recurrence is common, and 5-year survival is estimated at 8.2% [1]. Multiple chemotherapeutic regimens have been shown to extend survival in advanced PCa [29, 30], in particular the 2011 ACCORD-11 trial which reported improved survival with FOLFIRINOX compared to gemcitabine (11.1 versus 6.8 months). Metastasectomy has been well established in pancreatic neuroendocrine tumors, and in recent years, there have been a few small studies examining its utility in adenocarcinoma. Though literature has shown that pancreatectomy with synchronous hepatic metastasectomy can be performed safely [31–33], the long-term survival benefit is unclear. A 2017 systematic review and meta-analysis of 11 studies with 1147 patients concluded that there was a significant improvement in medium-term (< 3 years) survival compared to non-surgical approaches with median 1-year, 3-year, and OS of 40.9%, 13.3%, and 9.9 months, *p* < 0.0001 [34]. Pulmonary metastasectomy for PCa has also been described [35]. Arnaoutakis et al. [36] reported a significantly improved median OS (52 versus 22 months, *p* = 0.04) and survival after relapse (18.6 versus 7.5 months) for PCa undergoing resection for isolated pulmonary metastases. However, all of these individual studies were small and the patients highly selected. The authors recommended metastasectomy be considered only when an R0 resection can be achieved after pancreatectomy, the PCa has good tumor biology (seen by a favorable response to neoadjuvant chemotherapy), and the oligometastases are resectable [35]. To the best of our knowledge, there are, however, no studies in the literature examining the utility of surgical intervention in PC, even if R0 could be achieved. We report on the first case of CRS/HIPEC in PCa. Our one patient underwent a single CRS/HIPEC procedure for synchronous metastases. The PCI was 10 and CC-0 was achieved necessitating hepatectomy, colectomy, and diaphragmatic resection. The HIPEC agent used was mitomycin C. The patient received both neoadjuvant and adjuvant chemotherapy with gemcitabine. The patient did not experience perioperative morbidity. PFS and OS were 15 months.

### Hepatocellular carcinoma

Compared to pancreaticobiliary malignancies, relatively fewer patients with HCC (13–42%) have evidence of metastatic disease at diagnosis [37]. In unresectable HCC, the only effective systemic therapy is sorafenib, which offers significant OS benefits of up to 10.7 months, 2–3 months longer than with best supportive care [38]. However, the role of sorafenib in isolated PC without distant metastases is unclear. As such, there have been many studies examining the role of aggressive surgical therapy in patients with PC, with some promising results. Ding et al. conducted a literature review of 24 patients with PC from HCC treated with CRS and found 1-year and 2-year OS of 83% and 71%, respectively [39]. These findings were echoed by Lin et al. who reviewed 53 patients with HCC and PC and demonstrated significantly improved OS for CRS compared to best supportive care (12.5 versus 2.1 months, *p* = 0.0013) [40]. As an extension of this concept, CRS and HIPEC have been evaluated by a few authors [7, 10, 41]. Spiliotis et al. [41] reviewed 4 patients with localized PC who underwent CRS and HIPEC, with a mean PCI of 10.2. All patients received adjuvant sorafenib. The median PFS and OS were 13.5 and 30 months, respectively. In 2018, Mehta et al. published results from a multicenter study of 21 patients with HCC [11]. At the time of surgery, the median PCI was 14 and complete cytoreduction was achieved in 16 patients. The most frequent HIPEC agent used was cisplatin, followed by doxorubicin and mitomycin C. They did not comment on concomitant use of sorafenib. The median PFS and OS were 26.3 months and 46.7 months. Those patients who achieved complete cytoreduction had a significantly longer median OS (not reached) than those with incomplete cytoreduction (5.9 months), considerably longer than with sorafenib alone. A previous report from our institution by Tabrizian et al. described 14 patients with HCC and PC who were considered for CRS, of whom 7 also underwent HIPEC [10]. At the time of surgery, 6 were for metachronous disease and the median PCI was 12. Complete cytoreduction was possible in 6 patients, and the HIPEC agent used was mitomycin C in all cases. The median OS for all patients was 35.6 months, and they noted that the cohort with HIPEC had a higher OS (42.1 months) despite having higher PCI scores. This study reports on nine patients who underwent both CRS and HIPEC; 8 were for metachronous lesions. The median PCI was 12, and complete cytoreduction was achieved in 78% of patients. Two patients experienced major perioperative morbidity; one developed sepsis from pneumonia and required ICU stay, and the other died. Seven patients received adjuvant sorafenib. Median PFS and OS were 7 and 42 months, respectively.

When considering the role of CRS/HIPEC for HPB malignancies with PC, it is evident that these cancers behave very differently depending upon their primary tumor origin. However, current literature is very sparse. This study, similar to others, is underpowered due to the small sample size. The short follow-up period is also a limitation. Despite these drawbacks, there does appear to be evidence suggesting the utility of CRS in HCC with PC with an acceptable morbidity profile, provided complete cytoreduction can be achieved. Further survival benefit may be gained with the addition of HIPEC; more studies need to be conducted to better evaluate this. Perhaps, the most likely subset of patients to benefit from CRS/HIPEC would be those with ruptured HCC and presumed peritoneal dissemination, in whom primary hepatic resection may be otherwise contraindicated. The role of CRS/HIPEC in pancreaticobiliary malignancies is still unclear, though it could be considered in patients with limited PCI, resectable disease, and metachronous metastases with a longer disease-free interval. Future studies with longitudinal evaluation need to be conducted in order to better define its value in these settings.

## Conclusions

HPB malignancies with PC have poor survival with current palliative systemic therapies. CRS and HIPEC may offer a survival benefit for HCC with PC; however, there does not appear to be any benefit for pancreaticobiliary malignancies.

## Data Availability

The datasets generated during and/or analyzed during the current study are available from the corresponding author on reasonable request.

## Abbreviations

CC: Completeness of cytoreduction
CCa: Cholangiocarcinoma
CI: Confidence intervals
CRS: Cytoreductive surgery
EBL: Estimated blood loss
FLR: Future liver remnant
GBC: Gallbladder cancer
HR: Hazard ratios
HCC: Hepatocellular carcinoma
HPB: Hepatopancreaticobiliary
HIPEC: Hyperthermic intraperitoneal chemotherapy
IQR: Interquartile ranges
LOS: Length of stay
OS: Overall survival
PCa: Pancreatic adenocarcinoma
PCI: Peritoneal cancer index
PC: Peritoneal carcinomatosis
PFS: Progression-free survival
